# Bidirectional association between gastroesophageal reflux disease and depression: Two different nested case-control studies using a national sample cohort

**DOI:** 10.1038/s41598-018-29629-7

**Published:** 2018-08-06

**Authors:** So Young Kim, Hyung-Jong Kim, Hyun Lim, Il Gyu Kong, Miyoung Kim, Hyo Geun Choi

**Affiliations:** 1 Department of Otorhinolaryngology-Head & Neck Surgery, CHA Bundang Medical Center, CHA University, Seongnam, Korea; 2 Department of Otorhinolaryngology-Head & Neck Surgery, Hallym University College of Medicine, Anyang, Korea; 3 Department of Internal Medicine, Hallym University College of Medicine, Anyang, Korea; 4 Department of Laboratory Medicine, Hallym University College of Medicine, Anyang, Korea

## Abstract

The purpose of this study is to evaluate the associations between gastroesophageal reflux disease (GERD) and depression using a national sample cohort of the Korean population. Data were collected from individuals ≥20 years old in the Korean National Health Insurance Service-National Sample Cohort between 2002 and 2013. We designed two different nested case-control studies. In study I, 60,957 participants with depression were matched at a 1:4 ratio with 243,828 controls, and their previous histories of GERD were analyzed. In study II, 133,089 participants with GERD were matched at a 1:2 ratio with 266,178 controls, and their previous histories of depression were analyzed. Crude and adjusted odds ratios (ORs) were analyzed using unconditional logistic regression analyses, and 95% confidence intervals (CIs) were calculated. Subgroup analyses were performed according to age and sex. The adjusted OR for GERD was 2.01 (95% CI = 1.96–2.07) in the patients with depression (study I). The adjusted OR for depression was 1.48 (95% CI = 1.43–1.52) in the patients with GERD (study II). The results of the subgroup analyses were consistent. GERD and depression displayed bidirectional associations.

## Introduction

Gastroesophageal reflux disease (GERD) is one of the most common gastrointestinal disorders^1^. The upward movement of gastric contents into the esophagus causes troublesome symptoms and/or complications^1^. The prevalence of GERD is approximately 18.1–27.8% in North America, 8.8–25.9% in Europe, 2.5–7.8% in East Asia^2^, and 4.6–7.3% in Korea^3^. The pathogenesis of GERD is multifactorial. Hypotension of the lower esophageal sphincter, delayed acid clearance of the esophagus, and increased esophagogastric junction compliance have all been suggested as possible pathogenic factors^4^.

Depression is one of the most common diseases among humans^5^. The prevalence of depression is approximately 7.6% in the USA^6^ and 11% in the UK^7^. In Korea, the lifetime prevalence of major depressive disorder is 6.7% and has increased by 0.2% annually^8^. Depression influences various diseases, such as cardiovascular disease and diabetes mellitus^9^. Previous studies have reported an association between depression and GERD^10–13^. Anxiety or depression have been reported to increase the risk of GERD in some studies^14,15^, whereas other studies have suggested that GERD increases the risk of depression^10,12^. Some cross-sectional studies have also reported this association^11,13^; therefore, the direction of the effect is not clear. Verifying the direction of the association between GERD and depression is important because it may affect the approach to managing patients diagnosed with both diseases, as these patients are frequently treated in clinics.

The purpose of this study is to evaluate the associations between GERD and depression using a national sample cohort of the Korean population. Therefore, we designed two different nested case-control studies. In one study, we extracted data from patients with depression and their 1:4 matched controls (study I) and analyzed their previous histories of GERD. In the other study, we obtained data from patients with GERD and their 1:2 matched controls (study II) and analyzed their previous histories of depression.

## Results

### Study I

The rate of GERD was higher in the depression group (16.3% [9,959/60,957]) than in the control I group (9.0% [21,963/197,904], P < 0.001, Table 1). The general characteristics (age, sex, income, region of residence, and histories of hypertension, diabetes, and dyslipidemia) of the participants were exactly the same after matching (P = 1.000). Higher rates of ischemic heart disease and cerebral stroke were observed in the depression group (all, P < 0.05).

**Table 1:** General characteristics of the participants.

Characteristics	Study I (1:4 matching)			Study II (1:2 matching)		
	Depression (n, %)	Control I (n, %)	P-value	GERD (n, %)	Control II (n, %)	P-value
Age (years old)			1.000			1.000
20–24	3,851 (6.3)	15,404 (6.3)		4,526 (3.4)	9,052 (3.4)	
25–29	4,553 (7.5)	18,212 (7.5)		7,277 (5.5)	14,554 (5.5)	
30–34	5,243 (8.6)	20,972 (8.6)		10,098 (7.6)	20,196 (7.6)	
35–39	5,807 (9.5)	23,228 (9.5)		12,797 (9.6)	25,594 (9.6)	
40–44	6,176 (10.1)	24,704 (10.1)		16,652 (12.5)	33,304 (12.5)	
45–49	6,466 (10.6)	25,864 (10.6)		17,632 (13.2)	35,264 (13.2)	
50–54	6,208 (10.2)	24,832 (10.2)		17,674 (13.3)	35,348 (13.3)	
55–59	5,061 (8.3)	20,244 (8.3)		13,888 (10.4)	27,776 (10.4)	
60–64	4,740 (7.8)	18,960 (7.8)		11,943 (9.0)	23,886 (9.0)	
65–69	4,606 (7.6)	18,424 (7.6)		9,294 (7.0)	18,588 (7.0)	
70–74	3,861 (6.3)	15,444 (6.3)		6,380 (4.8)	12,760 (4.8)	
75–79	2,444 (4.0)	9,776 (4.0)		3,218 (2.4)	6,436 (2.4)	
80–84	1,294 (2.1)	5,176 (2.1)		1,249 (0.9)	2,498 (0.9)	
85+	647 (1.1)	2,588 (1.1)		461 (0.3)	922 (0.3)	
Sex			1.000			1.000
Male	20,776 (34.1)	83,104 (34.1)		60,503 (45.5)	121,006 (45.5)	
Female	40,181 (65.9)	160,724 (65.9)		72,586 (54.5)	145,172 (54.5)	
Income			1.000			1.000
1 (lowest)	886 (1.5)	3,544 (1.5)		3,087 (2.3)	6,174 (2.3)	
2	4,473 (7.3)	17,892 (7.3)		8,842 (6.6)	17,684 (6.6)	
3	4,202 (6.9)	16,808 (6.9)		8,859 (6.7)	17,718 (6.7)	
4	4,218 (6.9)	16,872 (6.9)		9,151 (6.9)	18,302 (6.9)	
5	4,760 (7.8)	19,040 (7.8)		10,008 (7.5)	20,016 (7.5)	
6	5,081 (8.3)	20,324 (8.3)		11,122 (8.4)	22,244 (8.4)	
7	5,414 (8.9)	21,656 (8.9)		12,716 (9.6)	25,432 (9.6)	
8	6,101 (10.0)	24,404 (10.0)		13,912 (10.5)	27,824 (10.5)	
9	7,017 (11.5)	28,068 (11.5)		16,163 (12.1)	32,326 (12.1)	
10	8,558 (14.0)	34,232 (14.0)		18,379 (13.8)	36,758 (13.8)	
11 (highest)	10,247 (16.8)	40,988 (16.8)		20,850 (15.7)	41,700 (15.7)	
Region of residence			1.000			1.000
Urban	27,882 (45.7)	111,528 (45.7)		61,912 (46.5)	123,824 (46.5)	
Rural	33,075 (54.3)	132,300 (54.3)		71,177 (53.5)	142,354 (53.5)	
Hypertension			1.000			1.000
Yes	22,530 (37.0)	90,120 (37.0)		47,429 (35.6)	94,858 (35.6)	
No	38,427 (63.0)	153,708 (63.0)		85,660 (64.4)	171,320 (64.4)	
Diabetes			1.000			1.000
Yes	12,125 (19.9)	48,500 (19.9)		24,412 (18.3)	48,824 (18.3)	
No	48,832 (80.1)	195,328 (80.1)		108,677 (81.7)	217,354 (81.7)	
Dyslipidemia			1.000			1.000
Yes	17,836 (29.3)	71,344 (29.3)		41,603 (31.3)	83,206 (31.3)	
No	43,121 (70.7)	172,484 (70.7)		91,486 (68.7)	182,972 (68.7)	
Ischemic heart disease			<0.001*			<0.001*
Yes	4,575 (7.5)	14,029 (5.8)		9,054 (6.8)	14,200 (5.3)	
No	56,382 (92.5)	229,799 (94.2)		124,035 (93.2)	251,978 (94.7)	
Cerebral stroke			0.013*			<0.001*
Yes	8,377 (13.7)	23,004 (9.4)		12,808 (9.6)	23,214 (8.7)	
No	52,580 (86.3)	220,824 (90.6)		120,281 (90.4)	242,964 (91.3)	
GERD			<0.001*			
Yes	9,959 (16.3)	21,963 (9.0)		N/A	N/A	
No	50,998 (83.7)	221,865 (91.0)		N/A	N/A	
Depression						<0.001*
Yes	N/A	N/A		7,625 (5.7)	10,474 (3.9)	
No	N/A	N/A		125,464 (94.3)	255,704 (96.1)	

*Chi-square test. Significance at P < 0.05.

GERD: gastroesophageal reflux diseases.

The crude and adjusted odds ratios (ORs) for GERD were 1.97 (95% confidence interval (CI) = 1.92–2.02) and 2.01 (95% CI = 1.96–2.07), respectively, in the depression group (P < 0.001, Table 2).

**Table 2:** Crude and adjusted ORs (95% CI) for GERD in patients with depression.

Characteristics	ORs of GERD			
	Crude	P-value	Adjusted^†^	P-value
Depression	1.97 (1.92–2.02)	<0.001*	2.01 (1.96–2.07)	<0.001*
Control	1.00		1.00	

*Logistic regression analyses. Significance at P < 0.05.

^†^Model adjusted for age, sex, income, region of residence, and histories of hypertension, diabetes, dyslipidemia, ischemic heart disease, and cerebral stroke.

In the subgroup analyses, higher crude and adjusted ORs for GERD were observed in the depression group (P < 0.05, Table 3). The adjusted ORs were 2.35 (95% CI = 2.10–2.63) in men <40 years old; 2.05 (95% CI = 1.91–2.21) in women <40 years old; 2.17 (95% CI = 2.03–2.33) in men 40–59 years old; 1.99 (95% CI = 1.90–2.09) in women 40–59 years old; 1.99 (95% CI = 1.85–2.15) in men ≥60 years old; and 1.90 (95% CI = 1.81–2.01) in women ≥60 years old.

**Table 3:** Subgroup analysis of crude and adjusted ORs (95% CI) for GERD in patients with depression stratified according to age and sex.

Characteristics	ORs for GERD			
	Crude	P-value	Adjusted^†^	P-value
Age <40 years old, men (n = 33,440)				
Depression	2.31 (2.07–2.59)	<0.001*	2.35 (2.10–2.63)	<0.001*
Control	1.00		1.00	
Age <40 years old, women (n = 63,830)				
Depression	2.05 (1.90–2.20)	<0.001*	2.05 (1.91–2.21)	<0.001*
Control	1.00		1.00	
Age 40–59 years old, men (n = 40,635)				
Depression	2.15 (2.01–2.30)	<0.001*	2.17 (2.03–2.33)	<0.001*
Control	1.00		1.00	
Age 40–59 years old, women (n = 78,920)				
Depression	1.98 (1.89–2.08)	<0.001*	1.99 (1.90–2.09)	<0.001*
Control	1.00		1.00	
Age ≥60 years old, men (n = 29,805)				
Depression	1.95 (1.81–2.09)	<0.001*	1.99 (1.85–2.15)	<0.001*
Control	1.00		1.00	
Age ≥60 years old, women (n = 58,155)				
Depression	1.88 (1.79–1.98)	<0.001*	1.90 (1.81–2.01)	<0.001*
Control	1.00		1.00	

*Logistic regression analyses. Significance at P < 0.05.

^†^Model adjusted for age, sex, income, region of residence, and histories of hypertension, diabetes, dyslipidemia, ischemic heart disease, and cerebral stroke.

### Study II

A higher rate of depression was observed in the GERD group (5.7% [7,625/133,089]) than in the control II group (3.9% [10,474/266,178], P < 0.001, Table 1). The general characteristics (age, sex, income, region of residence, and histories of hypertension, diabetes, and dyslipidemia) of the participants were exactly the same after matching (P = 1.000). Higher rates of ischemic heart disease and cerebral stroke were observed in the GERD group (all, P < 0.05).

The crude and adjusted ORs for depression were 1.48 (95% CI = 1.44–1.53) and 1.48 (95% CI = 1.43–1.52), respectively, in the GERD group (P < 0.001, Table 4).

**Table 4:** Crude and adjusted ORs (95% CI) for depression in patients with GERD.

Characteristics	ORs for depression			
	Crude	P-value	Adjusted^†^	P-value
GERD	1.48 (1.44–1.53)	<0.001*	1.48 (1.43–1.52)	<0.001*
Control	1.00		1.00	

*Logistic regression analyses. Significance at P < 0.05.

^†^Model adjusted for age, sex, income, region of residence, and histories of hypertension, diabetes, dyslipidemia, ischemic heart disease, and cerebral stroke.

In the subgroup analyses, higher crude and adjusted ORs for depression were recorded in the GERD group (P < 0.001, Table 5). The adjusted ORs were 1.44 (95% CI = 1.27–1.63) in men <40 years old; 1.64 (95% CI = 1.50–1.78) in women <40 years old; 1.51 (95% CI = 1.40–1.64) in men 40–59 years old; 1.47 (95% CI = 1.40–1.55) in women 40–59 years old; 1.39 (95% CI = 1.26–1.53) in men ≥60 years old; and 1.43 (95% CI = 1.34–1.52) in women ≥60 years old.

**Table 5:** Subgroup analysis of crude and adjusted ORs (95% CI) for depression in patients with GERD stratified according to age and sex.

Characteristics	ORs for depression			
	Crude	P-value	Adjusted^†^	P-value
Age <40 years old, men (n = 46,611)				
GERD	1.46 (1.29–1.65)	<0.001*	1.44 (1.27–1.63)	<0.001*
Control	1.00		1.00	
Age <40 years old, women (n = 57,483)				
GERD	1.65 (1.51–1.79)	<0.001*	1.64 (1.50–1.78)	<0.001*
Control	1.00		1.00	
Age 40–59 years old, men (n = 92,076)				
GERD	1.52 (1.40–1.64)	<0.001*	1.51 (1.40–1.64)	<0.001*
Control	1.00		1.00	
Age 40–59 years old, women (n = 105,462)				
GERD	1.49 (1.41–1.57)	<0.001*	1.47 (1.40–1.55)	<0.001*
Control	1.00		1.00	
Age ≥60 years old, men (n = 42,822)				
GERD	1.39 (1.26–1.53)	<0.001*	1.39 (1.26–1.53)	<0.001*
Control	1.00		1.00	
Age ≥60 years old, women (n = 54,813)				
GERD	1.44 (1.35–1.54)	<0.001*	1.43 (1.34–1.52)	<0.001*
Control	1.00		1.00	

*Logistic regression analyses. Significance at P < 0.05.

^†^Model adjusted for age, sex, income, region of residence, and histories of hypertension, diabetes, dyslipidemia, ischemic heart disease, and cerebral stroke.

## Discussion

Higher ORs for GERD were observed in the depression group. Moreover, the ORs for depression were higher in patients with GERD. Because the general characteristics of the extracted study population were different in study I and study II, the ORs (OR for GERD in patients with depression; OR for depression in patients with GERD) were not the same in study I and study II. In both studies, these relationships were consistent in the subgroup analyses stratified according to age and sex. To our knowledge, this study is the first to confirm the bidirectionality of these associations using two different cohorts. Because the two diseases are associated, clinicians treating one disease should consider the presence of the other disease.

The results of this study were similar to those of previous studies. The incidence ratio for depression was 2.29 (95% CI = 1.58–3.36) in patients with GERD in a previous study^10^, and another study revealed that the hazard ratio for depression was 3.37 (95% CI = 2.49–4.57) in patients with GERD^12^. Here, the adjusted OR for GERD in patients with depression was 2.01 (95% CI = 1.96–2.07) in study I. Previous studies have reported ORs of 1.7 (95% CI = 1.4–2.1)^13^ and 3.16 (95% CI = 2.71–3.68)^11^ for GERD in patients with depression. Here, the adjusted OR for depression in patients with GERD was 1.48 (95% CI = 1.43–1.52) in study II.

We have identified several possible explanations for the observation that GERD increased the risk of depression. First, the esophageal mucosa of patients with GERD contains large amounts of cytokines, such as interleukin (IL)-6, IL-8, IL-1beta, interferon gamma (IFN-γ), and tumor necrosis factor alpha (TNF-α)^16^. The increased levels of these immune mediators in peripheral organs might be associated with the upregulation of central nervous system inflammation^17^, which might affect depression and bipolar disorder^18,19^. Second, frequent arousal by GERD might activate the autonomic nervous system and increase sympathetic activation^20^. Acid reflux stimulates the vagus nerve and triggers bronchoconstriction^21^, which could result in sleep disorders and affect mood disorders^22^. Third, the reflux symptom itself could result in depression if patients are constantly feeling upset about their condition^23^.

Depression might also increase the risk of GERD. First, the fear of reflux symptoms might increase the individual’s perception of reflux symptoms^23^. Psychological factors could reduce the sensation threshold in the body^23^ and increase the sensation of esophageal stimulation^24^. Second, depression might actually increase reflux. Psychological factors can decrease the pressure of the lower esophageal sphincter^23^, change esophageal motility^23^, increase gastric acid secretion^24^, and decrease acid clearance in the esophagus^24^. Third, antidepressant medication might aggravate reflux^25^. Anticholinergic drugs may lower the pressure of the lower esophageal sphincter^26^. Moreover, drugs have been shown to delay gastric emptying, inhibit esophageal peristalsis, and decrease salivary secretion^25^.

Furthermore, confounders such as obesity, eating habits, smoking, alcohol drinking, lack of sleep, and high stress might aggravate both GERD and depression^27,28^. Stress induces GERD^29^ and promotes depression^30^.

The advantages of this study are similar to those of our previous studies using the national sample cohort^31–33^. We used a large, representative nationwide population. Because the NHIS data include all citizens of the nation without exception, no participants were lost to follow-up. The control groups were randomly selected by matching for age, sex, income, region of residence, and past medical histories to avoid confounding effects. Furthermore, an adjusted regression model was used to minimize the influence of confounders. Because of the large number of participants, we maintained the statistical power in the split study design. Thus, we performed subgroup analyses. Moreover, we performed two different studies. Because of the cohort study design, we could differentiate whether GERD or depression might initiate the relationship.

This study has several limitations. Despite the cohort study design, we were not able to exclude the effects of possible confounders that might affect both GERD and depression. Because data on body mass index, smoking, alcohol, and sleep habits were not available, we could not adjust for these factors. We were not able to analyze the severity of both GERD and depression, and patients who did not consult a clinician may have been missed. Because of the nested case-control study design, the ORs between risk factors (independent variable) and results (dependent variable) should be interpreted carefully. Because the participants were selected using ICD-10 codes, this study has the possibility of over- or underestimation of the associations examined. We considered GERD on the basis of diagnostic codes and treatment histories without endoscopic findings. Therefore, we were not able to determine whether these patients had only symptomatic GERD or erosive GERD.

The ORs for GERD were higher in patients with depression, and higher ORs for depression were also observed in patients with GERD. These associations were consistent among different age groups and sexes.

## Materials and Methods

### Study Population and Data Collection

This study was approved by the ethics committee of Hallym University (2014-I148). The Institutional Review Board excused the requirement to obtain written informed consent from study participants. Every survey reported in this study was conducted in agreement with the guidelines of the ethics committee of Hallym University.

The present study was based on data from the Korean National Health Insurance Service-National Sample Cohort (NHIS-NSC). To minimize nonsampling errors, the samples were extracted exactly from the mother population of the Korean NHIS. Approximately 2% (one million) of the whole Korean population (50 million) was chosen. These sampled data represent the mother population obtained by stratified systemic sampling methods. The selected population was classified at 1,476 levels (age [18 categories], sex [2 categories], and income level [41 categories]). The validity of the sample cohort was confirmed in a previous study^34^. The specific procedures for sampling the cohort are described on the website of the National Health Insurance Sharing Service^35^. This cohort database comprises detailed medical management histories for every participant from 2002 to 2013: (i) personal information, (ii) health insurance claim codes (procedures and prescriptions), (iii) diagnostic codes using the International Classification of Disease-10 (ICD-10), (iv) death records from the Korean National Statistical Office (using the Korean Standard Classification of Disease), (v) socio-economic data (residence and income), and (vi) medical examination data

The precise population statistics were derived using these NHIS data. The Korean Health Insurance System is mandatory for all Koreans. Using the Health Insurance Review & Assessment (HIRA) system, all medical records can be traced without exception in Korea. Moreover, the possible overlapping of medical records was minimized, even when a patient moved to a different region of residence. All patients are registered using a 13-digit registration number in Korea. The 13-digit registration number is legally issued to every Korean for life. The death of a patient must be reported to an administrative entity before a funeral is conducted. The date and cause of death are listed on a death certificate issued by medical doctors.

### Participant Selection

All 1,125,691 patients with 114,369,638 medical claim codes were used in this study. Among these participants, patients with depression were included. Depression was diagnosed based on the ICD-10 codes F31 (bipolar affective disorder) through F39 (unspecified mood disorder) by a psychiatrist from 2002 through 2013^33,36^. Only participants who were treated ≥2 times were included (n = 68,019).

GERD was selected using the ICD-10 code K21 during the same periods considered for depression^37^. Patients who were treated ≥2 times and were prescribed a proton pump inhibitor (PPI) for ≥2 weeks were included (n = 137,807).

#### Study I

**Figure 1:**
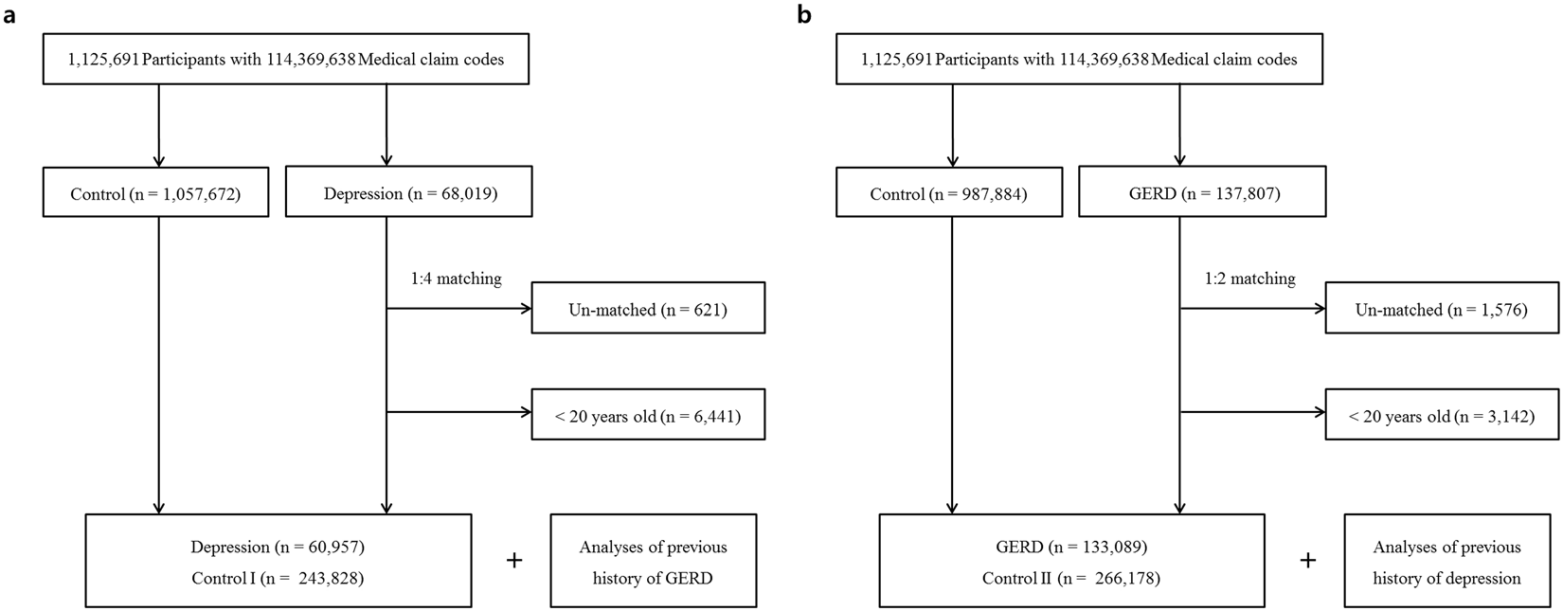
A schematic illustration of the participant selection process used in the present study. (**a**) Of the 1,125,691 total participants, 60,957 participants with depression were matched with 243,828 control I participants for age, group, sex, income group, region of residence, and past medical histories. (**b**) Of the 1,125,691 total participants, 133,089 participants with GERD were matched with 266,178 control II participants for age, group, sex, income group, region of residence, and past medical histories.

The participants in the control I group, who were never treated for depression during the same period, were matched 1:4 with the participants with depression for age, group, sex, income group, region of residence, and past medical histories (hypertension, diabetes, and dyslipidemia). These participants were extracted from the mother population (n = 1,057,672). The participants in the control I group were arranged using a random number order and then chosen in descending order. The control I participants and participants with depression were presumed to be concurrently enrolled in this study (index date). Thus, participants who died before the index date were excluded from the control group. Participants with depression for whom we were not able to identify a sufficient number of matching participants were excluded (n = 621). Participants aged less than 20 years were also excluded (n = 6,441). Ultimately, 60,957 participants with depression participants and 243,828 control I participants were included (Fig. 1a). However, the past medical histories of ischemic heart disease or cerebral stroke were not matched because such rigorous matching elevated the dropout rate due to the absence of control participants. Using these matched groups, we investigated the previous histories of GERD in both the depression and control I groups.

#### Study II

The participants in the control II group, who were never diagnosed with GERD, were matched 1:2 with the GERD participants from 2002 through 2013 in this cohort. The control groups were extracted from the mother population (n = 987,884). The age, group, sex, income group, region of residence, and past medical histories (hypertension, diabetes, and dyslipidemia) were matched between the participants with GERD and the control II group. The control II participants were arranged using another set of random numbers and then chosen in descending order to minimize selection bias. The control II participants and participants with depression were presumed to be concurrently enrolled in this study (index date). Thus, participants who died before the index date were excluded. The participants with GERD for whom we were not able to identify a sufficient number of matching participants were excluded (n = 1,576). Participants aged less than 20 years were also excluded (n = 3,142). As a result, 133,089 participants with GERD and 266,178 control II participants were included (Fig. 1b). However, the past medical histories of ischemic heart disease and cerebral stroke were not matched for the same reason mentioned above. The previous histories of depression in both the GERD and control II groups were analyzed.

### Variables

Age groups were categorized in 5-year intervals: 20–24, 25–29, 30–34…, and 85+ years old. Fourteen age groups were classified. The income groups were initially classified into 41 classes (one health aid class, 20 self-employment health insurance classes, and 20 employment health insurance classes). These groups were then integrated into 11 classes (class 1 [lowest income]-11 [highest income]). Region of residence was classified into 16 areas in accordance with administrative areas. These regions were further classified into urban (Seoul, Busan, Daegu, Incheon, Gwangju, Daejeon, and Ulsan) and rural (Gyeonggi, Gangwon, Chungcheongbuk, Chungcheongnam, Jeollabuk, Jeollanam, Gyeongsangbuk, Gyeongsangnam, and Jeju) areas.

The ICD-10 codes were used to identify the past medical histories. For a precise diagnosis, participants who were treated ≥2 times for hypertension (I10 and I15), diabetes (E10-E14), and dyslipidemia (E78) were included in these categories. Ischemic heart disease (I24 and I25) and cerebral stroke (I60-I66) were included if the participants were treated ≥1 time.

### Statistical analyses

An unconditional logistic regression analysis was conducted to analyze the ORs for GERD in the patients with depression in study I. Another unconditional logistic regression analysis was conducted to analyze the ORs for depression in patients with GERD in study II. Crude (simple) and adjusted (age, sex, income, region of residence, and histories of hypertension, diabetes, dyslipidemia, ischemic heart disease, and cerebral stroke) models were employed, and 95% CIs were determined.

Subgroup analyses were performed according to age and sex (<40 years old, 40–59 years old, and ≥60 years old; men and women).

Two-tailed analyses were used, and P values less than 0.05 were defined as indicating a statistically significant difference. The SPSS v. 22.0 software was used for statistical analyses (IBM, Armonk, NY, USA).
